# Body Image and Sociocultural Predictors of Body Image Dissatisfaction in Croatian and Chinese Women

**DOI:** 10.3389/fpsyg.2020.00731

**Published:** 2020-05-06

**Authors:** Ivana Stojcic, Xiawei Dong, Xiaopeng Ren

**Affiliations:** ^1^Key Laboratory of Behavioral Science, Institute of Psychology, Chinese Academy of Sciences, Beijing, China; ^2^Department of Psychology, University of Chinese Academy of Sciences, Beijing, China

**Keywords:** culture, body image dissatisfaction, thin-ideal internalization, China, Croatia

## Abstract

In the current paper, two different studies were designed to investigate body image dissatisfaction and perception of the attractive female body in Chinese and Croatian women and men using the correlational and experimental study research approach. Study 1 comprised 266 Chinese (160 women; 106 men) and 261 Croatian participants (161 women; 100 men). Women from both countries were asked to complete the measures of the Contour Rating Scale, SATAQ – 4, Body Area Scale and demographic data, while men were asked to complete the measures of the Contour Rating Scale and demographic data via online surveys. The obtained results indicated that thin internalization predicted body image dissatisfaction in both samples. Besides, while both samples scored relatively high on thin-ideal internalization, Chinese women, who were also in general less satisfied with their body image, had higher scores. Chinese women also scored higher on musculature internalization and felt more pressure from family, friends, and media to conform to standard beauty norms compared to Croatian women. The samples also differed in their perceptions of preferable body size, where Chinese women had a more negative perception of their actual body size. The obtained results furthermore revealed that Chinese men preferred thinner women compared to Croatian men. Moreover, both samples misjudged what their female counterparts found attractive, thinking that women wanted to have even thinner bodies than they actually reported. Similarly, women from both cultures revealed a preference for thinner figures than the ones selected as the most attractive by the opposite sex. Finally, in Study 2, experimental manipulation of thin-ideal was used to examine whether thin priming affected body image dissatisfaction. The obtained results revealed that when primed with thin-ideal women from both countries felt lower satisfaction with their body size. The observed effect was stronger for Chinese participants. Limitations of the current study are discussed in the conclusion.

## Introduction

Previous studies have shown that body dissatisfaction and excessive investment in the body are precursors to the full range of unhealthy eating behaviors, including binge eating, restrictive dieting, and self-induced vomiting (Thompson and Stice, 2001; Wertheim et al., 2001; Levine and Piran, 2004). Extreme preoccupation with weight and body shape have been identified as vital risk factors for the development of eating disorders and have been included as diagnostic criteria for both anorexia and bulimia (Lydecker et al., 2017). Furthermore, body image dissatisfaction has been associated with depression, stress, low self-esteem (Johnson and Wardle, 2005), increased negative mood (Tiggemann and McGill, 2004), reduced appraisal of one’s own physical appeal (Bessenoff, 2006), global shame (Hayaki et al., 2002), increased social anxiety (Cash et al., 2004), emotional distress, appearance rumination, and a tendency toward unnecessary appearance management (Paxton et al., 2006; Etu and Gray, 2010).

### Tripartite Influence Model

One of the most commonly used theories to explain the development of body image dissatisfaction is the Tripartite Influence Model (Thompson et al., 1999). This model proposes that three influences (peers, parents, and media) affect body image and eating problems through two mediational mechanisms: internalization of societal standards of appearance and excessive appearance comparison. According to this theory, social influences, such as peers, family, and media pressure, individuals to adhere to culturally defined standards of beauty (Stice and Agras, 1998; Grabe et al., 2008). After persistent exposure to these messages, the ideal becomes part of individuals’ personal beliefs of what constitutes attractiveness and beauty, and individuals start to internalize the appearance ideals, which they eventually interpret as originating from within the self. Those that internalize the dominant message within their socio-cultural environment to a greater extent, but at the same time don’t feel as they meet the standards are at higher risk of body image dissatisfaction (Thompson and Stice, 2001; Cafri et al., 2005). According to previous studies, thin-ideal internalization is an essential concept for a complete comprehension of body image dissatisfaction (Thompson and Stice, 2001; Stice, 2002). Previous studies have shown that the thin-ideal internalization is directly related to body dissatisfaction (Stice and Agras, 1998) and eating disorder symptomatology (Ballentine and Ogle, 2005; Suisman et al., 2012). Furthermore, thin internalization was also found to mediate the relationship between sociocultural influences and body dissatisfaction (Tiggemann, 2003; Keery et al., 2004). Musculature internalization is equivalent of thin internalization for male body image dissatisfaction (Schaefer et al., 2015). Although male body image has received considerably less research attention, existing studies have revealed that internalization of the muscular ideal is significantly associated with increased body dissatisfaction, negative affect, and unhealthy behaviors aimed at increasing muscle size (Karazsia and Crowther, 2008; Thompson et al., 2012). Nonetheless, over recent years a new body image trend of muscular and fit female bodies has become increasingly popular in Western societies (Bozsik et al., 2018; Rodgers et al., 2018), thus suggesting that musculature internalization might also be applicable in the female population. A substantial amount of previous research has provided support for Tripartite Influence Model, especially among young girls and women (e.g., Carlson Jones, 2004; Keery et al., 2004; Cafri et al., 2005; Bedford and Johnson, 2006; Shroff and Thompson, 2006). Besides body image disturbances and disordered eating, this model was also a good fit in explaining various body management practices among women like tanning and exercising, as well as attitudes toward cosmetic surgery (Sharp et al., 2014). The Tripartite Influence Model was also supported among boys and men (Karazsia and Crowther, 2008; Grammas and Schwartz, 2009; Jackson and Chen, 2010; Tylka, 2011; Hazzard et al., 2019); as well as among populations of homosexual and bisexual orientations (Tylka and Andorka, 2012; Huxley et al., 2015), and among various non-Western countries (Yamamiya et al., 2008; Mellor et al., 2009; Jackson and Chen, 2010; Papp et al., 2013; Shagar et al., 2019).

Not that long ago, it was generally believed that the body image dissatisfaction was predominantly Western World phenomenon (Nasser et al., 2003; Grabe and Hyde, 2006); nevertheless, the turn of the 21^st^ century marked onset of the overwhelming spread of body image dissatisfaction in numerous countries outside the Western World (Rathner, 2001; Nasser et al., 2003). Tripartite influence model is especially relevant to non-White populations, since these women may feel under pressure to achieve standards of beauty that may significantly deviate from their ethnic features. Previous studies have found that when non-white women judge themselves through Western beauty standards, they are at higher risk of developing body image dissatisfaction and eating disorders (Hall, 1995; James et al., 2001; Oney et al., 2011). For example, a study conducted in Fiji revealed that body image dissatisfaction among young girls substantially increased following the introduction of western television to the island (Becker, 2004). Similar trends were observed in other non-Western countries such as Japan (Pike and Borovoy, 2004), Malaysia (Mellor et al., 2009), Korea (Lee, 2000), India (Shroff and Thompson, 2004), and many more (Swami et al., 2010). On the other hand, some studies have also reported higher resistance to thin-ideal internalization in women of African American heritage (Wildes et al., 2001). This same effect was also reported for Latina women, whose ethnic identity protected them to a certain extent from negative body image and negative comparisons with westernized beauty ideals (Schooler, 2008). Conversely, Asian cultures have been reported to be very susceptible to body image dissatisfaction and disturbances (Jung and Lee, 2006). Women in these countries have endorsed new canons of beauty that significantly differ from their traditional features and that include extremely thin bodies (Han, 2003; Rongmuang et al., 2011). Some Asian countries have developed a preference for large eyes and high noses, which notably deviate from their ethnic features, thus perpetuating the unprecedented spread of the cosmetic industry in these countries (Jung and Lee, 2006).

There is an interesting correspondence between increases in body image dissatisfactions in Asia and increases in body image dissatisfactions in the former socialist countries in Europe (Jung and Forbes, 2007). As it used to be the case with Asian countries, and the rest of the non–Western world, it is believed that body image dissatisfaction had a very low occurrence among the former socialistic countries in Europe (Catina and Joja, 2001; Rathner, 2001). Some researchers argue that socialist systems promoted gender equality, rejection of gender stereotypes, and preconceived notions of femininity and normative physical appearance (Catina and Joja, 2001). The demise of the socialist systems drastically redefined the women’s role within these societies possibly leading toward the alteration of beauty ideals and the rise of body image dissatisfaction. In fact, the rise of eating disorders in Western Europe and the United States, which occurred in the second half of the 20^th^ century, coincided with a number of sweeping changes such as the rise of the consumer economy and associated impact on the status of women (Gordon, 2001). It has been well-documented that the prevalence of eating disorders was greater in industrialized or “post-industrialized” countries such as the United States, Western Europe, Japan, and similar compared to pre-industrialized societies such as those in sub-Sharan Africa (Levine and Smolak, 2010). Feminist theories argue that cultures with strong patriarchal tradition, rigid gender roles, and very fast social transformation, including fast-growing opportunities for women, are especially likely to internalize unrealistic appearance standards and to observe relatively high levels of body image dissatisfaction (Paludi, 2010).

### Social Norms and Cultural Values

To fully apprehend the body image in two different cultures, it is necessary to understand the social and cultural norms, i.e., rules or expectations of behavior and thoughts based on shared beliefs on gender-roles within these specific cultural groups (Strahan et al., 2006). Attitudes toward gender-roles imply the general perception of gender roles such as gender-related tasks and power distribution. Since people are socialized into their community’s gender ideologies and rules about how men and women are supposed to think and behave, they accept these norms at face value. These values and norms can directly affect the way power are distributed in society, which in most of the patriarchal societies, is typical to the disadvantage of women (Sen and Östlin, 2008; Marcus and Harper, 2014). Consequently, the male view of femininity and beauty becomes not only a preference but a standard to strive toward. For example, traditional gender roles associate femininity with beauty and concern with appearance (Lennon et al., 1999). Indeed, previous studies have found that among young women, the notion of femininity is positively related to the importance of appearance. This finding suggests that an attractive appearance is likely to be more important for women endorsing the traditional attitudes toward gender roles than to women who hold non-traditional attitudes (Green et al., 2008). Thus, a woman’s traditional attitudes about male-femalee relations may implicitly require that she possesses and displays the physical and behavioral characteristics she believes men find attractive. Inglehart et al. (2002) reported that as countries undergo industrialization and modernization processes, their views on gender equality become more liberal compared to that of residents in less developed countries (Inglehart and Baker, 2000). Existing research has also identified a relationship between gender equality and cultural value dimensions of individualism/collectivism, power distance, and masculinity/femininity, which strongly determine the levels of gender equality in a specific society. E.g., individualistic cultures tend to have higher levels of gender equality compared to collectivistic cultures because the sanctity of the person in such individualistic cultures prevails over the ascribed status or social roles (Cohen and Kitayama, 2007). Previous studies have shown that gender roles were more pronounced in countries that scored higher on the cultural dimension of power distance, which describes the extent to which people in the society accept the uneven distribution of power (Basabe and Ros, 2005). Societies that score high on this dimension presume men to be competitive and to strive for material success and women to serve and care for non-material aspects of life and for children. In more masculine societies, gender inequality dominates, the social roles of sexes are different, and the mother has a weaker position in the family. Contrary, more feminine societies offer both sexes, especially women, more opportunities for the fulfillment of multiple social roles that are associated with more well-being and relationship satisfaction (Costa et al., 2001).

### Body Image Dissatisfaction in Transition Economies

Both countries of our interest, China and Croatia, had a long history of communist and socialist social organizations, respectively, and have been witnessing fast socio-economic transition over the past couple of decades. Furthermore, both countries have strong patriarchal traditions rooted in Confucianism (Leung, 2003) and Catholic Church (Ramet and Matic, 2007), respectively; as well as heritage in a collectivist tradition that is gradually shifting toward more individualistic values (Podrug et al., 2006; Steele and Lynch, 2013; Hamamura and Xu, 2015; Su et al., 2016). Existing research proves that while religiosity does appear to promote patriarchal ideas (Hunnicutt, 2009; Ogle and Batton, 2009), it can also promote healthy body image and discourage dieting behaviors by supplying a source of worth other than the body (Brumberg, 2000; Kim, 2006), unlike Confucianism that has traditionally linked the worth of a woman with her corporality, i.e., the ability to produce domestic labor and to carry and give birth to sons thus perpetuating the male line. It has been suggested that even after the modernization, women in traditional Confucius cultures are encouraged to value themselves through their corporeal bodies, which are no longer respected for the ability to bear children but for the physical beauty that can positively affect one’s career or marital prospects (Kim, 2003). This has also been supported by recent research on the consumption of cosmetic surgery in China, which revealed that motivations to undertake plastic surgery operations came primarily from Chinese cultural hierarchies, i.e., family and society, as well as from the need to compete in a modernizing society (Hua, 2009). Confucianism, among others, endorses the idea of filial piety, which requires children to respect, support, and regard elderly in the family, as well as teachers, professional superiors, or anyone who is older in age. In such a culture, the family is the building block of society, while this hierarchical system of respect also applies to one’s country values as well (Chan, 2003). Previous studies have shown that perceived norms of more proximal or relevant reference groups may have a stronger impact on individual behaviors for members of such groups, including one’s gender (Lewis and Neighbors, 2004). Consequently, women’s identity is constructed in terms of social roles or identities where the physical appearance gets interpreted through group expectations and as something that can potentially bring face or dishonor on the family (Lindridge and Wang, 2008). As far as Croatia is concerned, according to existing research that has compared Croatian women with women from two other former Yugoslav countries, Slovenia and Serbia, on body image and beautifying practices, Croatian women have shown to be more likely to engage in physical beauty practices compared to their counterparts, and they have shown to be the most inclined toward cosmetic surgery. They were also the ones who have endorsed the post-traditional values to the highest level (Čevnik, 2016).

Culture has an impact on numerous factors that contribute to body image, including the media, peers, and family socialization (Grabe et al., 2008; Bell and Dittmar, 2011; Ferguson et al., 2011; Jones, 2011; Levine and Chapman, 2011; McCabe and McGreevy, 2011; Abraczinskas et al., 2012). Compared to the body of knowledge on body image dissatisfaction in Western World countries, there is still relatively little knowledge about these same concerns in non-Western countries. In words of Cash and Pruzinsky (2002) there is obvious paucity in “comparative cross-cultural studies of body image that are crucial to enhance our understanding of the diversity of body images and the influence of culture on body image development, dysfunction, and change” (p. 513). A large number of existing studies on body image dissatisfaction have focused on comparing non-Western populations with Western ones (e.g., Bush et al., 2001; Jung and Lee, 2006; Jung et al., 2010; Taylor et al., 2013); yet, there are far fewer studies on body image dissatisfaction among two or more non-Western samples. The latter approach might be useful to move beyond established socio-cultural influences underpinning the body image dissatisfaction toward more specific and culturally driven interpretations of the same. The purpose of the present research was to overcome this limitation and examine body image dissatisfaction and perception of the attractive female body in Chinese and Croatian women and men using the correlational and experimental study research approaches. Two principal hypotheses were considered across the two studies, namely that thin internalization would predict body image dissatisfaction in both countries, and that both Chinese and Croatian women would display overall high levels of body image dissatisfaction; however, these levels would be higher for Chinese women. We designed two different studies to test our hypotheses. Study 1 investigated body image dissatisfaction in both samples, and influence of family, peers, and media on body image dissatisfaction, as well as male perspective on thin ideal in both countries. In Study 2, we experimentally manipulated the thin ideal in women from both countries to make causal inferences between thin internalization and body image dissatisfaction, thus overcoming the limitations related to the correlational nature of Study 1.

## Study 1

Study 1 examined peer, family, and media influences on body image dissatisfaction in women from China and Croatia, as well as the importance of individual body items in each cultural context. In Study 1, we also examined men’s preferences for female body size in both cultures.

As already stated, both countries of our interest have witnessed a fast socio-economic transition in the past couple of decades (International Monetary Fund, Monetary, and Capital Markets Department, 2000). Both countries also have strong patriarchal traditions rooted in Confucianism (Leung, 2003) and Catholic Church (Ramet and Matic, 2007), where former emphasizes social and family hierarchy thus placing women in a subordinate position, and former advocates the ideas of equality, thus placing women in a more equal position to men. Furthermore, both countries are experiencing greater than ever interaction with Western cultural values (Leung, 2003). Separate researches from China and Croatia report increase in desire for thinness (Leung et al., 2001; Bralic and Kovacic, 2005; Rukavina and Pokrajac-Bulian, 2006; Chen et al., 2007) and increase in dieting and disordered eating among adolescent girls and young women (Pokrajac-Bulian, 1998; Pokrajac-Bulian et al., 2006; Jackson and Chen, 2008). Likewise, increased facial appearance dissatisfaction (Jackson and Chen, 2008), as well as height dissatisfaction, have been reported in the Chinese population (Watts, 2004; Chen et al., 2006). Also, recent research has proved that people in very wealthy and westernized countries of Asia had very high levels of body image dissatisfaction (BID; Holmqvist and Frisén, 2010), which is the effect we expected to observe in Chinese sample given the strong Chinese economic progression and a gradual shift toward individualistic values (Wu, 2004; Steele and Lynch, 2013). While the similar socio-economic transition is taking place in Croatia, Croatian women resemble more closely imposed Eurocentric beauty ideals widely represented by the Western media, which we believe could have a somewhat beneficial effect on their overall levels of BID in comparison to China.

Furthermore, previous studies have shown that one reason heterosexual women may internalize the thin-ideal is that thin women represented in the media tend to be rewarded by men (Fouts and Burggraf, 2000; Greenberg et al., 2003). Accordingly, a woman’s body image is strongly associated with her perception of what she thinks men prefer (Meltzer and McNulty, 2015). Numerous studies worldwide have reported that men have an overall preference for women with lower waist to hip ratio, which at the physiological level, has been associated with higher estrogen and progesterone levels (Jasienska et al., 2004), and thus higher possibility for conception (Jones et al., 2005). Consequently, the male perspective on desirable female size might greatly shape the general opinion on what is understood as physically attractive. This might be even more prominent in cultures with strong patriarchal traditions (Leung, 2003; Ramet and Matic, 2007), where men hold the primary power and have predominant roles in societies. Previous studies have identified an association between gender equality and Hofstede’s cultural value dimensions of individualism/collectivism, power distance, and masculinity-femininity, which strongly determine how gender-egalitarian a particular society is likely to be. China scores higher on power distance and masculinity and lowers on individualism compared to Croatia, which suggests that gender inequalities are higher in China compared to Croatia^1^. This is also consistent with the data reported by the United Nations and the gender inequality index, according to which China was on the 85^th^ place for gender inequalities and Croatia on 46^th2^. Accordingly, our aim was to examine to which extent thin women were seen as more attractive in two countries of our interest. Specific hypotheses for Study 1 include:

(H1): In both countries, higher levels of thin internalization will predict higher body image dissatisfaction.
(H2): Chinese women will have higher levels of thin and musculature internalization, as well as higher levels of family, peer, and media pressure compared to Croatian women.
(H3): Chinese women will be more dissatisfied with their body image compared to Croatian women.
(H4): Chinese men will prefer thinner women than their Croatian counterparts.

### Methods

#### Participants

The participants in this study were 321 women: 160 Chinese women aged between 18 and 30 years (*M* = 26.34, *SD* = 2.70); and 161 Croatian women aged between 18 and 30 years (*M* = 24.16, *SD* = 2.27), respectively, and 206 men: 106 Chinese men aged between 19 and 30 years (*M* = 26.48, *SD* = 2.68); and 100 Croatian men aged between 18 and 30 years (*M* = 22.17, *SD* = 3.18), respectively.

#### Procedure

Participants were recruited using a snowball sampling method, and they were invited to fill out online surveys posted at two online survey platforms: Survey Monkey^3^ for Croatian participants and Wenjuanxing^4^ for Chinese participants. Women were asked to fill out the following standardized instruments: Contour Rating Scale, Body Area Scale, and Sociocultural Attitudes Toward Appearance Questionnaire-4, as well as to provide their demographic data related to age, height, weight, and nationality, while men were asked to fill out Contour Rating Scale and to provide their demographic data. The Chinese and Croatian versions of the measures used in this study were translated as part of this study through formal procedures of translation and back-translation (Brislin, 1970; Song et al., 2019). The English version was used as a template from which the translation/back-translation was conducted for the Chinese and Croatian versions. Here we introduced the procedure using the Chinese version. The procedure of translation/back-translation in this study was conducted by the expert committee. Two translation teams were formed. Each translation team consisted of three native Chinese individuals who were proficient in both English and Chinese languages. The three translators in the first team made their translations independently, and then discussion ensued until a consensus was reached for the first drafts of the Chinese versions of the measures. The three translators in the second team made back-translation drafts independently and then made a final decision on the back-translation versions. Next, an expert committee was formed with a faculty member and a graduate student from the psychology department of the Institute of Psychology, Chinese Academy of Sciences. All committee members were familiar with the questionnaires. Based on the comparison of the back-translation version and the original English version, and consideration of cultural adaptation, the expert committee made a decision on the final Chinese versions of the measures. The only difference for the Croatian version is that the committee members were made up of the first author and other two assistants proficient in both Croatian and English language.

The study was presented to participants as a study on the influence of the socio-cultural environment on body image. It took around 15 min for women to finish the survey and 5 min for men. By completing the survey, the participants have provided consent to participate in the research.

#### Measures

##### Contour rating scale – (CDRS; Thompson and Gray, 1995)

CDRS comprises nine drawings of a female figure. The figures gradually increase in size from extremely thin (1) to very obese (9). Participants are requested to rate the ideal figure, i.e., the one that reflects the best the way they ideally want to look (CDRSw2) and their current size, i.e., the one that closest resembles the way they perceive themselves (CDRSw1). The difference between the current size and ideal reproduces the body size dissatisfaction index (CDRSwscore) were requested to (a) choose the figure they perceived as the most attractive (CDRS1m); (b) choose the figure they thought most women find the most attractive and wish to have (CDRS2m) and (c) to choose the figure they thought was most widely represented in the media (CDRS3m). The previous study reported 14-week test–retest reliabilities ranging from 0.71 to 0.90, as well as satisfactory construct and discriminant validity (Wertheim et al., 2004). We slightly modified the figure drawings in a way that we removed lines picturing the ribs from drawings 1–4, and we obscured the women’s face and hair features with black squares (see “Supplementary Material”).

##### Body area scale – (BAS; Lerner et al., 1973)

BAS is an assessment system used for evaluating satisfaction with one’s body weight, body size, and satisfaction with different body parts. It contains 24 body parts or attributes rated on a five-point Likert scale ranging from “very satisfied” to “very dissatisfied.” Body dissatisfaction is calculated by determining the number of body parts with which an individual expresses dissatisfaction. Dissatisfaction is determined by a response of “moderately dissatisfied” or “very dissatisfied.” Previous research has reported satisfactory reliability of this scale, as well as considerable “conceptual overlap” with other body image measures (Richards et al., 1990). In the present study, BAS has demonstrated excellent internal consistency, with Cronbach’s alpha of 0.88 in the Chinese sample and 0.89 in the Croatian sample.

##### Sociocultural attitudes toward appearance questionnaire-4 – (SATAQ-4; Schaefer et al., 2015)

SATAQ-4 has a total of 22 items that are divided across a five-factor structure containing the subscales of: (1) Thin Internalization: Thin/Low Body Fat subscale that contains five items and assesses to what degree a respondent internalizes a thin body with low body fat as an ideal. Example items include: I want my body to look very thin/I think a lot about having very little body fat. (2) Muscular Internalization: Muscular/Athletic subscale that contains five items and assesses to what degree a respondent internalizes an athletic body with muscles as an ideal. Example items include: I think a lot about looking muscular/I spend a lot of time doing things to look more muscular. (3) Family Pressures: Family subscale that contains four items and assesses to what degree a respondent experiences pressure from family to look a certain way. Example items include: I feel pressure from family members to look thinner/Family members encourage me to decrease my level of body fat. (4) Peer Pressures: Peers subscale that contains four items and assesses to what degree a respondent feels pressure from peers to look a certain way. Example items include: My peers encouraged me to get thinner./I get pressure from my peers to decrease my level of body fat. (5) Media Pressures: Media subscale that contains four items and assesses to what degree a respondent feels pressure from the media to look a certain way. Example items include: I feel pressure from the media to look thinner/I feel pressure from the media to improve my appearance. SATAQ-4 has already been used in Chinese (Jung and Forbes, 2007) and Croatian populations (Horak, 2008). In the present study, the SATAQ-4 subscales have demonstrated good to excellent internal consistency, with following Cronbach’s alphas: 0.60, 0.84, 0.88, 0.84, 0.95 for thin internalization, musculature internalization, family, peer and media pressure, respectively in Croatian sample; 0.81, 0.90, 0.79, 0.85, 0.86 for thin internalization, musculature internalization, family, peer and media pressure, respectively in a Chinese sample.

##### Body mass index – (BMI)

The self-reported height and self-reported weight were used to calculate the BMI in participants using the following formula: weight/height2.

### Statistical Analysis

Statistical Package for Social Sciences (SPSS) version 20 was used for data analysis (IBM; United States). First, we performed correlation analyses, which were followed by regression analyses and independent-sample *t*-tests for female samples. Afterward, group comparisons for men were conducted using independent sample *t*-tests, which were followed by 2 × 2 ANOVA. The detailed results are reported in Tables 1–6.

**TABLE 1:** Correlation matrix for Chinese sample.

	***M***	***SD***	**1**	**2**	**3**	**4**	**5**	**6**	**7**	**8**
(1) Age	26.34	2.70								
(2) BMI	21.12	4.75	0.09							
(3) Family	2.87	0.90	0.228**	0.03						
(4) Peers	3.28	0.97	0.15	0.09	0.479**					
(5) Media	3.33	0.94	0.11	0.08	0.461**	0.864**				
(6) TI	4.26	0.57	0.14	0.09	0.164*	0.311**	0.312**			
(7) MI	2.74	0.93	0.183*	–0.05	0.232**	0.168*	0.171*	0.01		
(8) CDRSw score	1.33	1.59	–0.03	0.232**	0.08	0.294**	0.334**	0.447**	–0.1	

N = 160; TI, thin internalization; MI, musculature internalization. *Correlation is significant at the 0.05 level (two-tailed). **Correlation is significant at the 0.01 level (two-tailed).

**TABLE 2:** Correlation matrix for Croatian sample.

	***M***	***SD***	**1**	**2**	**3**	**4**	**5**	**6**	**7**	**8**
(1) Age	24.16	2.27								
(2) BMI	21.99	3.46	0.11							
(3) Family	1.88	1.04	0.04	0.556**						
(4) Peers	1.59	0.79	–0.03	0.347**	0.503**					
(5) Media	2.86	1.38	0.02	0.320**	0.243**	0.437**				
(6) TI	3.17	0.77	0.05	0.443**	0.294**	0.329**	0.544**			
(7) MI	2.50	0.85	–0.09	0.067	0.142	0.252	0.166	0.294**		
(8) CDRSw score	1.07	1.45	0.08	0.698**	0.404**	0.366**	0.523**	0.688**	0.15	

N = 161; TI, thin internalization; MI, musculature internalization. *Correlation is significant at the 0.05 level (two-tailed). **Correlation is significant at the 0.01 level (two-tailed).

**TABLE 3:** Comparison of sociocultural predictors of body image dissatisfaction between two cultural groups.

	***Chinese***		***Croatian***				
	***M***	***SD***	***M***	***SD***	***T-Test***	***p***	***d***
Thin internalization	4.26	0.57	3.17	0.76	−14.43	0.00	1.79
Musculature internalization	2.74	0.93	2.50	0.85	−2.38	0.05	0.28
Family pressure	2.87	0.90	1.88	1.03	−17.09	0.00	1.04
Peer pressure	3.29	0.97	1.60	0.79	−5.72	0.00	2.09
Media pressure	3.32	0.93	2.86	1.38	−3.49	0.00	0.41

*SATAQ-4 is graded with five point Likert scale; higher scores indicate higher agreement.*

**TABLE 4:** Multiple hierarchical regressions with body image dissatisfaction as assessed by CDRS scale as the dependent variable.

**Block**	***F* (df)**	**Adj. *R*^2^**	**Item**	**β**	***t***	***p***	***F* (df)**	**Adj. *R*^2^**	**Item**	**β**	***t***	***p***

	***Croatian***						***Chinese***					
1	74.94* (2, 158)	0.48	Age	0.01	0.1	0.92	4.69** (2, 156)	0.05	Age	–0.05	–0.69	0.492
			BMI	0.69	12.16	<0.001			BMI	0.24	3.04	< 0.01
2	47.25* (7, 153)	0.66	Age	0.01	0.1	0.92	9.56* (7, 151)	0.28	Age	–0.10	–1.36	0.177
			BMI	0.48	8.1	<0.001			BMI	0.18	2.69	<0.01
			Family	–0.02	–0.41	0.686			Family	–0.06	–0.81	0.422
			Peer	0.02	0.35	0.725			Peer	–0.01	–0.05	0.961
			Media	0.15	2.67	<0.01			Media	0.26	1.94	0.055
			TI	0.40	6.71	<0.001			TI	0.38	5.20	< 0.001
			MI	–0.03	–0.55	0.583			MI	–0.11	–1.51	0.133

*TI, thin internalization; MI, musculature internalization.*

**TABLE 5:** Independent Samples *t* -tests between BAS items in two cultural groups.

	***Chinese***		***Croatian***				
	***M***	***SD***	***M***	***SD***	***t -Test***	***p***	***d***
Facial complexion	3.48	1.13	2.29	1.21	−9.12	0.00	1.03
Ears	3.93	0.86	1.65	1.05	−21.29	0.00	2.51
Chest	3.11	1.18	2.40	1.24	−5.21	0.00	0.61
Profile	3.31	0.96	2.61	1.19	−5.72	0.00	0.69
Weight_	2.79	1.16	2.78	1.31	−0.09	0.93	0.00
Eyes	3.70	1.07	1.57	0.87	−19.55	0.00	2.35
Height_	3.16	1.26	1.82	1.04	−10.39	0.00	1.21
Ankle	3.43	0.94	1.86	1.06	−14.02	0.00	1.65
Waist	2.91	1.18	2.45	1.36	−3.28	0.00	0.38
Arms	3.08	1.13	2.48	1.24	−4.56	0.00	0.52
Legs	2.86	1.23	2.84	1.30	−0.13	0.89	0.01
General appearance	3.40	0.93	2.43	1.04	−8.80	0.00	1.01
Hips	3.16	1.08	2.42	1.21	−5.79	0.00	0.66
Shoulder	3.19	1.04	2.16	1.14	−8.52	0.00	0.97
Mouth	3.43	1.07	1.86	1.08	−13.11	0.00	1.57
Neck	3.43	0.93	1.81	0.93	−15.59	0.00	1.80
Teeth	2.99	1.13	2.47	1.26	−3.91	0.00	0.45
Nose	3.23	1.08	2.51	1.30	−5.37	0.00	0.62
Chin	3.28	1.02	2.02	1.12	−10.46	0.00	1.19
Hair texture	3.21	1.10	2.05	1.21	−8.95	0.00	1.00
Body built	3.07	0.95	2.33	1.16	−6.26	0.00	0.73
Hair color	3.70	0.94	1.50	0.73	−23.39	0.00	2.72
Thighs	2.82	1.22	2.68	1.26	−1.03	0.31	0.11
Face	3.29	1.00	2.11	1.08	−10.20	0.00	1.18

*BAS is graded with five point Likert scale; higher scores on individual items indicate higher dissatisfaction.*

**TABLE 6:** Comparison of Chinese and Croatian men’ view on female body size.

	***Chinese men***		***Croatian men***				
	***M***	***SD***	***M***	***SD***	***T-Test***	***p***	***d***
CDRS1m	3.67	1.21	4.22	1.03	−3.516	0.00	0.49
CDRS2m	2.73	1.43	3.17	1.01	−2.562	0.01	0.36
CDRS3m	3.17	1.51	3.01	1.19	0.837	0.40	0.12

*CDRS1m- figure men perceived as the most attractive; CDRS2m – figure men thought most women find the most attractive and wish to have; CDRS3m- figure men thought was most widely represented in the media.*

### Results

Chinese women (*M* = 26.34, *SD* = 2.70) were older compared to Croatian women (*M* = 24.16, *SD* = 2.27), *F*(1,319) = 61.63, *p* < 0.001. Furthermore, BMI revealed no statistical difference in BMI among Croatian women (*M* = 21.99, *SD* = 3.46) and Chinese women (*M* = 21.12, *SD* = 4.75); *F*(1,318) = 3.57, *p* = 0.06. Descriptive statistics for all variables and bivariate correlations were also calculated for each sample independently and are reported in Tables 1, 2. Chinese men (*M* = 26.48, *SD* = 2.68) were older than Croatian men (*M* = 22.17, *SD* = 3.18), *F*(1,215) = 111.19, *p* < 0.001, while there was no difference in BMI between Croatian (*M* = 23.96, *SD* = 2.83) and Chinese men (*M* = 22.99, *SD* = 5.11); *F*(1,205) = 48.02, *p* = 0.09.

Next, independent samples *t*-tests were run to examine the potential differences between the internal and external pressures in both countries. Culture was used as the independent variable and thin internalization, musculature internalization, family pressure, media pressure and peer pressure as dependent variables. The independent-samples *t*-test revealed that Chinese women scored higher on thin internalization (4.26 ± 0.57) compared to Croatian women (3.17 ± 0.76), *t*(319) = −14.43, *p* < 0.001. They also scored higher on musculature internalization [(Chinese vs. Croatian = 2.74 ± 0.93 vs. 2.50 ± 0.85), *t*(319) = −2.38, *p* < 0.05], family pressure [(Chinese vs. Croatian = 2.87 ± 0.90 vs. *M* = 1.88 ± 1.03), *t*(319) = −17.09, *p* < 0.001], media pressure [(Chinese vs. Croatian = 3.32 ± 0.93 vs. *M* = 2.86 ± 1.38), *t*(319) = −3.49, *p* < 0.001], and peer pressure [(Chinese vs. Croatian = 3.29 ± 0.97 vs. *M* = 1.60 ± 0.79), *t*(319) = −5.72, *p* < 0.001]. Corresponding effect size is presented in Table 3.

To test which of the variables predicted body dissatisfaction assessed by the CDRS scale, we conducted a multiple hierarchical regression where age and BMI were included in the first block as predictor variables given that previous studies have identified these variables as relevant biological predictors in body image (Ålgars et al., 2009; Slevec and Tiggemann, 2011), while SATAQ-4 subscale scores were entered as predictor variables in the second block.

The regression results for the Croatian sample showed that age and BMI explained 48% of the variance, while SATAQ-4 variables accounted for 18% of the variance. In the final model, we found that BMI (β = 0.48, *p* < 0.001), Thin Internalization (β = 0.40, *p* < 0.001) and Media pressure (β = 0.15, *p* < 0.01) significantly predicted body image dissatisfaction assessed by CDRS scale. The regression results for the Chinese sample showed that age and BMI explained 5% of the variance, while SATAQ-4 variables accounted for 23% of the variance. In the final model, we found that BMI (β = 0.18, *p* < 0.01), and Thin Internalization (β = 0.38, *p* < 0.001) significantly predicted body image dissatisfaction assessed by CDRS scale (see Table 4).

Next, an independent-samples *t*-test was conducted to compare average body image dissatisfaction assessed by the BAS scale in two groups of women. The obtained results revealed a significant difference between Chinese (*M* = 12.33, *SD* = 5.74) and Croatian women (*M* = 4.70, *SD* = 4.15); *t*(319) = −13.65, *p* < 0.001. These results suggested an obvious difference in body image dissatisfaction assessed by the BAS scale between the two groups of women, where Croatian women were, on average, dissatisfied with four to five aspects of their physical appearance, while Chinese women were dissatisfied with 12 to 13 body areas. The independent samples *t*-tests were also run to examine the differences between the body image dissatisfaction assessed by the BAS scale with individual body parts in both countries. Statistically significant differences observed between two cultural groups on individual BAS items and their corresponding *t*-values and effect sizes are shown in Table 5.

When analyzing individual BAS scale items, it was found that Chinese women expressed high dissatisfaction with almost all body parts and were especially dissatisfied with their ears, eyes, and hair color. They also reported relatively high levels of dissatisfaction with their mouth, neck, ankles, and general appearance. Contrary, Croatian women revealed to be the most dissatisfied with their legs, weight, and the looks of their thighs. Interestingly, both Chinese (*M* = 2.79, *SD* = 1.16) and Croatian women (*M* = 2.78, *SD* = 1.31) were almost equally dissatisfied with their weight.

**FIGURE 1:**
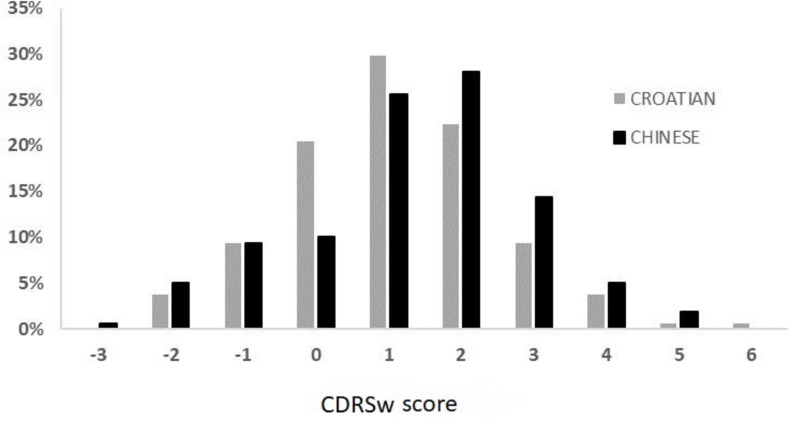
Contour Rating Scale score percentages in both populations. A positive score indicates a preference for a slimmer body.

This discontent with body weight observed in both samples was furthermore supported by the body image dissatisfaction index (CDRS scale). The independent-samples *t*-test that was used to compare the CDRSw scores between the two groups revealed no statistically significant differences, thus suggesting that both Croatian (*M* = 1.07, *SD* = 1.45) and Chinese women (*M* = 1.33, *SD* = 1.59) were equally dissatisfied with their body size; *t*(319) = −1.50, *p* = 0.132. However, the perception of ideal body size was significantly different among the two groups, with Chinese women (*M* = 3.32, *SD* = 1.17) preferring significantly thinner bodies compared to Croatian women (*M* = 3.81, *SD* = 0.96); *t*(319) = 4.16, *p* < 0.001. Next, we examined the figures that the participants from both cultures selected from the CDRS scale as their current and their ideal figures. Among the examined samples, only 10% of Chinese women and 20.5% of Croatian women were completely satisfied with their body size, whereas 75% of Chinese women and 66.5% of Croatian women felt their body was bigger than their perceived ideal size. Figure 1 illustrates the trends in dissatisfaction with body size among the two examined groups.

The independent samples *t*-test was also conducted in order to examine to which extent Chinese and Croatian men preferred thin to heavier women, which body size they thought was the most appealing to women, and which body size they thought was most represented in the media. The observed results suggested an obvious difference in body size preferences among Chinese and Croatian men, where Chinese men preferred thinner women (*M* = 3.67, *SD* = 1.21) compared to Croatian men (*M* = 4.22, *SD* = 1.03). Chinese men also selected significantly smaller body size as the one that women from their country would prefer (*M* = 2.73, *SD* = 1.43) compared to their Croatian counterparts (*M* = 3.17, *SD* = 1.01). The results are shown in Table 6.

Next, an additional analysis was performed by combing the men’s preference and women’s preference so as to explore the effect of gender and culture on desirable body size. A 2 (culture: China, Croatia) x 2 (gender: men, women) ANOVA was performed on the influence of culture and gender on the perception of the idealized body. First, we wanted to test the relationship between the body size most attractive to men (CDRS1m), and the one women rated as the most attractive (CDRSw2). Besides the significant main effect of culture on the perception of idealized body *F*(1,522) = 28.87, *p* < 0.001, the main effect of gender *F*(1,522) = 18.03, *p* < 0.001 was also significant, while the interaction between gender and culture was not. These results suggested that both Chinese (*M* = 3.32, *SD* = 1.17) and Croatian women (*M* = 3.81, *SD* = 0.96) wanted body size that was much thinner than the one chosen by men as desirable one (*M* = 3.67, *SD* = 1.20; *M* = 4.22, *SD* = 1.02, respectively).

Next, we wanted to examine the relationship between the body size men thought women want (CDRS2m) and the one women rated as the most attractive (CDRSw2). Again, besides the significant main effect of culture on the perception of idealized body *F*(1,521) = 27.64, *p* < 0.001, the main effect of gender was also significant *F*(1,521) = 36.46, *p* < 0.001, while the interaction between gender and culture was not. These results suggested that when comparing the body size men thought women from their countries might desire to actual preferences reported by Chinese (*M* = 3.32, *SD* = 1.17) and Croatian women (*M* = 3.81, *SD* = 0.96), men from both countries substantially misjudged the body size that women found attractive, opting for very small body size (*M* = 2.73, *SD* = 1.43; *M* = 3.17, *SD* = 1.01, respectively).

### Discussion

In Study 1, we tested a series of hypotheses revealing that thin internalization was related to body image dissatisfaction assessed by the CDRS scale in both Chinese and Croatian samples. When accounting for other variables identified as relevant to body image dissatisfaction by previous studies (Bearman et al., 2006; Laus et al., 2011; Santana et al., 2013), thin internalization was still the strongest predictor variable. Furthermore, while women from both countries reported having a stronger preference for slimmer bodies compared to their current body size, this discrepancy, as well as more obvious preference for slimmer body size, was more prominent in a Chinese sample. Chinese women scored significantly higher on thin internalization compared to their Croatian counterparts, and they had significantly lower levels of satisfaction with almost all individual body parts assessed by the BAS scale compared to Croatian women, which was entirely consistent with our hypotheses.

Additionally, the obtained results revealed that women from both cultures wanted thinner bodies than the ones selected as the most attractive by men, which was consistent with previous studies (Swami et al., 2010). This was in line with evolutionary theory, according to which men view of the attractive body is an important determinant of body image satisfaction among heterosexual women (Bergstrom et al., 2004). Interestingly enough, men from both countries agreed on the female body size most commonly represented in media, which suggests that both Chinese and Croatian men started from a similar baseline, i.e., both cultural contexts were exposed to similar media images endorsing a similar desirable female size. Even so, Chinese men preferred thinner women compared to Croatian men, thus confirming our hypothesis. As already stated, gender inequalities are greater in Chinese culture compared to Croatian culture that suggests that Chinese, in general, hold a more traditional view of gender roles (Cohen and Kitayama, 2007), which also encompass the ideas of desirable female body image. If we consider that Asian women, in general, have thinner bodies compared to European women, it is possible that exposure to thin-ideal in such culture results in even more extreme pursuit of thinness, which is further reinforced by male-dominant hierarchical social structures.

## Study 2

Study 1 showed that thin internalization was the strongest predictor of body image dissatisfaction among all the examined sociocultural influences and that male and female perception of desirable body size were significantly inconsistent, with women opting for significantly thinner body size compared to the one chosen by men in both countries. However, Study 1 was correlational, which means that causal relationships between thin internalization and body image dissatisfaction could not be inferred. In order to overcome this limitation, we experimentally manipulated thin exposure in women from both cultural groups. The specific hypothesis for Study 2 was that women in the experimental group from both countries would experience higher body image dissatisfaction following thin ideal priming, but the observed effect would be stronger among Chinese women.

### Methods

#### Participants

The participants in this study were 200 women: 100 Chinese women aged between 18 and 30 years (*M* = 22.85, *SD* = 3.69), and 100 Croatian women aged between 18 and 30 years (*M* = 22.19, *SD* = 2.56), respectively. Participants were recruited through printed and electronic advertisements posted on different notice boards across countries of interest. They were randomly allocated to one of the groups (50 allocated to experimental; and 50 allocated to control group).

#### Procedure

The stimulus materials containing images of professional fashion models were drawn from two popular fashion outlets (Vogue and Yoka). A pilot test was conducted with a small group of female volunteers (*N* = 10; five from each cultural group) to ensure that the fashion models were considered attractive and thin by the desired populations (18 to 30 years). Photos of 30 models who had the highest attractiveness ratings were included in the study. All the photos were full-page (A4-size) pictures, and they featured a full view of a woman. Control advertisements featured various products, and they did not contain any persons or body parts (see “Supplementary Material”). Participants were informed that they were participating in an experiment on consumer behavior. After receiving the instructions, participants were asked to complete a demographic questionnaire and then to rate each of the 30 advertisements using the following statements:” In my opinion, this ad was effective./In my opinion this ad was ineffective.” The purpose of this measure was to make the cover story more believable and to encourage participants to pay closer attention to presented advertisements. To furthermore distract the participants from the real purpose of the study, additional marketing questions were created by the investigator. After viewing all of the images, participants completed all the questions, including the CDRS scale. The whole procedure took approximately 15 min, after which the participants were debriefed about the actual purpose of the study.

#### Measures

##### Contour rating scale – (CDRS; Thompson and Gray, 1995)

We used a somewhat modified version of the CDRS scale, where nine drawings of a female figure were substituted with images of virtual models created by modelmydiet website. The figures were modified to look more closely virtual models that can be found in online stores with virtual dressing rooms or shopping applications (e.g., Looklet, H&M), thus making the cover story more believable (see “Supplementary Materials”). The figures gradually increased in size from extremely thin (1) to very obese (9). Participants were requested to rate the ideal figure, i.e., the one that reflects the best the way they ideally want to look (CDRS2) and their current size, i.e., the one that closely resembles the way they perceive themselves (CDRS1). The difference between the ideal and current size reproduces the body size dissatisfaction index.

#### Statistical Analysis

Statistical Package for Social Sciences (SPSS) version 20 was used for all statistical analyses. We conducted a series of 2 × 2 ANOVA analyses to compare groups across cultures and conditions. Detailed results are reported in Tables 7, 8.

**TABLE 7:** Descriptive Statistics for evaluation of body image size by condition and culture.

		***China***		***Croatia***	
		***M***	***SD***	***M***	***SD***
CDRS1	Control group	4.43	1.49	4.54	1.46
	Experimental group	4.32	1.39	4.48	1.61
CDRS2	Control group	3.35	1.01	3.64	0.92
	Experimental group	2.52	0.71	3.54	0.97
CDRS score	Control group	1.08	1.34	0.90	1.50
	Experimental group	1.8	1.03	0.94	1.17

*CDRS1- current size; CDRS2 – ideal body size; CDRS score – discrepancy between CDRS1 and CDRS2; China: control group *N* = 49; experimental group = N; Croatia: *N* = 50 both groups.*

**TABLE 8:** Group condition x Culture Factorial Analysis of Variance for the perception of current body size, ideal body size, and body image dissatisfaction.

		***df***	***MS***	***F***	***p.***	**η^2^**
CDRS1						
	CULTURE	1	0.92	0.41	0.52	0.00
	CONDITION	1	0.35	0.16	0.69	0.00
	CULTURE * CONDITION	1	0.03	0.01	0.91	0.00
CDRS2						
	CULTURE	1	21.44	25.89	0.00	0.12
	CONDITION	1	10.69	12.90	0.00	0.06
	CULTURE * CONDITION	1	6.57	7.93	0.01	0.04
CDRS score						
	CULTURE	1	13.49	8.35	0.00	0.04
	CONDITION	1	7.15	4.43	0.04	0.02
	CULTURE * CONDITION	1	5.72	3.54	0.06	0.02

*CDRS1- current size; CDRS2 – ideal body size; CDRS score – discrepancy between CDRS1 and CDRS2. R Squared = 0.003 (Adjusted R Squared = −0.012). R Squared = 0.194 (Adjusted R Squared = 0.182). RR Squared = 0.078 (Adjusted R Squared = 0.063). MS = Mean squares, effect size = η^2^ or partial η^2^.*

### Results

A 2 (culture: China, Croatia) x 2 (condition: experimental, control) analysis of variance was conducted on the influence of culture and thin exposure on the perception of current body, idealized body and body image dissatisfaction calculated as the discrepancy between the former two (see Tables 7, 8).

As shown in Table 8, the main effect of culture *F*(1,195) = 0.41, *p* = 0.52, or thin exposure *F*(1,195) = 0.16, *p* = 0.69 on perception of current body size, i.e., CDRS1, were not significant, nor was their interaction; *F*(1,195) = 0.01, *p* = 0.91. This was in line with our expectations, given that priming of the thin ideal was less likely to change the general idea one has of their actual size compared to body size one desires to have.

Regarding the perception of ideal body size, i.e., CDRS2, statistical analyses yielded completely opposite results to CDRS1, such that there was a significant interaction effect between culture and thin exposure *F*(1,195) = 7.93, *p* < 0.01. Also, the main effects of culture *F*(1,195) = 25.89, *p* < 0.001, and condition were both statistically significant *F*(1,195) = 12.90, *p* < 0.001; thus suggesting that experimental manipulation had a significant effect on the perception of the ideal body size among women assigned to experimental groups in both countries, where they expressed stronger preference toward thinner body size compared to control groups.

Finally, regarding the CDRS score, i.e., the discrepancy between the ideal and preferred body size, the interaction effect was not significant *F*(1,195) = 3.54, *p* < 0.06; while both main effects had significant effect on CDRS score, whereas women from China (*M* = 1.44, *SD* = 0.13) reported significantly higher levels of CDRS score compared to Croatian women (*M* = 0.92, *SD* = 0.13) *F*(1,195) = 8.35, *p* < 0.001; while women exposed to experimental manipulation (*M* = 1.37, *SD* = 0.13) *F*(1,195) = 4.43, *p* < 0.04 reported significantly higher levels of CDRS score compared to those from control groups (*M* = 0.99, *SD* = 0.13).

### Discussion

The obtained results confirmed that exposure to thin-ideal leads to higher levels of body image dissatisfaction in women regardless of their cultural identity, which is in line with existing research on thin internalization and body image dissatisfaction (e.g., Thompson and Stice, 2001; Stice, 2002; Tiggemann, 2003; Keery et al., 2004; Thompson et al., 2004; Keel and Forney, 2013). The reported results also provided support for our hypothesis, according to which Chinese women would experience higher levels of body image dissatisfaction, which was also in line with existing research arguing that Asian women are very susceptible to body image dissatisfactions (e.g., Han, 2003; Jung and Lee, 2006; Holmqvist and Frisén, 2010; Rongmuang et al., 2011), and that Asian countries are more prone to body image dissatisfactions compared to European countries (Holmqvist and Frisén, 2010).

## General Discussion

The current study aimed to examine how Croatian and Chinese women and men felt about female attractiveness, as well as to understand the influence of cultural background on body image satisfaction in women. Overall, the results of the current study supported our hypotheses, showing that Chinese women had higher levels of musculature and thin internalization, where the latter one predicted body image dissatisfaction in both samples. Chinese women also felt more pressure from family, peers, and media to conform to standard beauty norms compared to Croatian women. The samples also differed in their perceptions of preferable body size, where Chinese women had a more negative perception of their actual body size. Furthermore, women from both cultures revealed a preference for thinner figures than the ones selected as the most attractive by the opposite sex. Similarly, Chinese men reported preferring women of smaller size compared to Croatian men, even though men from both countries were in agreement on the female body size most widely represented in the media, which further suggests that both cultural contexts were exposed to similar thin-ideal media messages. Finally, the experimental manipulation revealed that thin exposure led to higher body image dissatisfaction in women from both cultures; however, this effect was more obvious in Chinese women. These findings are reviewed in more detail below.

Our results showed that the thin internalization strongly predicted body image dissatisfaction in both countries, being the strongest predictor of body image dissatisfaction in Chinese women, and the second strongest predictor in Croatian women. These findings are in line with a number of previous studies that have shown how thin internalization is the risk factor for body image dissatisfaction (Keel and Forney, 2013). They are also consistent with the premise that the thin-ideal internalization is the essential concept for complete comprehension of body image dissatisfaction (Fitzsimmons-Craft et al., 2012), and compared to the awareness of socio-cultural standards or mere exposure to idealized media images, it has the stronger impact on body image dissatisfaction (Cafri et al., 2005). Previous studies have shown that not all women respond to idealized media images and imposed sociocultural standards in the same way (Joshi et al., 2004), and those women who internalize the prevalent socio-cultural attitudes about the desirable appearance are at the higher risk of developing body image dissatisfaction (Thompson and Stice, 2001). Additionally, our results revealed significant differences between Chinese and Croatian women on thin internalization, musculature internalization, family pressure, media pressure, and peer pressure, whereas Chinese women reported being more susceptible to all of the above. Cultural context and ethnicity have been shown to have a protective effect against body image dissatisfaction (Warren et al., 2005); nonetheless, Asian cultures appear to be very susceptible to body image dissatisfaction (Jung and Lee, 2006), which was consistent with our results. This could be explained by the construct of collectivism, which includes psychological concepts such as attitudes, values, self-representations, and social contexts (Triandis, 2001). Brewer and Chen (2007) have introduced an aspect of collectivism that differentiates collectivism into relational collectivism and group collectivism. Relational collectivism refers to the relationship and role that individual has with regard to others, where individuals achieve a balance between the expression of social conformity and individuality that culturally differs in relation to specific relational or more symbolic collective contexts. According to this approach, Westerners achieve collectivism through group collectivism, whereas Easterners tend to rely on relational collectivism (Kreuzbauer et al., 2009). As was previously mentioned, China has its collectivist cultural traditions rooted in Confucianism (Leung, 2003), while in Croatia, these traditions stem from the long-lasting influence of the Catholic Church (Ramet and Matic, 2007). Accordingly, religious affiliation falls under group collectivism, whereas Confucianism is more deeply ingrained in Chinese social life and thus is more likely to affect a person’s identity (Huang and Gove, 2015). In cultures with strong relational collectivism, relationships with other members of the group and the interconnectedness between people have a central role in a person’s identity. Existing studies have shown that perceived norms of more proximal or relevant reference groups may have a stronger impact on individual behaviors for members of such groups, including one’s gender (Lewis and Neighbors, 2004), which in turn may affect perceptions of group-specific body image norms. These observations were especially interesting in relation to musculature internalization. Originally, musculature internalization SATAQ subscale was used for the male population as body image concerns in male populations mainly involve a desire for greater muscularity (Cafri et al., 2002; Thompson and Cafri, 2007). Given that muscular and fit female bodies have become increasingly popular in Western societies’ over recent years (Bozsik et al., 2018; Rodgers et al., 2018), we assumed this same trend might be slowly spreading outside the Western countries, which was supported by our study results. Since Chinese women appeared to be very susceptible to thin internalization, it was an educated guess that they will also have a stronger tendency toward endorsing new body ideals such as musculature or fit ideal. In fact, one study showed that both Chinese men and women rated male and female Caucasian models as more attractive than Chinese models (Jankowiak et al., 2008), thus highlighting the role of the media in transmitting Western appearance ideals to non-Western societies, as well as the extent of media’s influence in such contexts (e.g., Becker, 2004; Frith and Gleeson, 2004). Western cultural values instruct that appearance is vital to women’s value and role in society, and that thinness guarantees success and life satisfaction (Warren et al., 2005). In non-Western contexts, women would have to strive even harder to meet the preset ideals, given that modification of certain physical features goes beyond simple dieting, thus suggesting that body image would be more complex concept related to other physical features besides body fat, which was supported by our results but also by previous studies (Jackson and Chen, 2008; Tewari and Alvarez, 2009). Accordingly, in the present study, Chinese women appeared to be less satisfied with all of the physical items addressed in the study. These results were also in line with previous studies that have shown how people from affluent and westernized Asian countries are more dissatisfied with their bodies than people from the United States, while Americans are less happy with their bodies than Europeans (Holmqvist and Frisén, 2010). Furthermore, Chinese women were especially dissatisfied with their ears, eyes, and hair color. Contrary, Croatian women revealed to be most dissatisfied with their legs, weight, and the looks of their thighs. The dissatisfaction with facial features and hair has been previously reported as very relevant factors in overall body image satisfaction in Asian women (Tewari and Alvarez, 2009). In addition, separate researches from both countries have reported an increase in desire for thinness (Leung et al., 2001; Bralic and Kovacic, 2005; Rukavina and Pokrajac-Bulian, 2006; Chen et al., 2007) and increase in dieting and disordered eating among adolescent girls and young women (Rukavina and Pokrajac-Bulian, 2006). Likewise, increased facial appearance dissatisfaction (Jackson and Chen, 2008), as well as height dissatisfaction in China, have also been reported (Chen et al., 2006). A somewhat surprising finding was the overall satisfaction with individual body sites observed in the Croatian sample. While the observed differences did provide full support for our hypothesis that was based on the fact that Croatian women resemble more closely imposed Eurocentric beauty ideals widely represented by the Western media, the discontent in the Croatian sample was unexpectedly small. In fact, it appears that the Croatian sample perfectly fits the thin ideal paradigm based on the normative discontent with body size (Littleton and Ollendick, 2003) whereas body image dissatisfaction appears to be a more complex concept in a Chinese sample. It should also be noted that Chinese people tend to be more negative in self-evaluations (Sedikides et al., 2003, 2005), as well as more self-critical (Heine et al., 2000), which could also potentially explain the observed differences, and thus needs to be examined by future studies.

Interestingly, both Chinese and Croatian women were almost equally dissatisfied with their body size; however, the perception of ideal body size was significantly different between the two groups, with Chinese women reporting higher preferences for thinner bodies compared to Croatian women. Further analysis revealed that among the examined samples, only 10% of Chinese women and 20.5% of Croatian women were completely satisfied with their body size, whereas 75% of Chinese women and 66.5% of Croatian women felt their body was bigger than their perceived ideal size. These results provided further support for the overwhelming endorsement of thin-ideal through industrial and developing countries (Leavy, 2004). In the present study, we did not use the Western sample, where most of the thin ideals appear to originate from; nevertheless, according to a study that has compared body image dissatisfaction in the United States, Ukraine, and Ghana using the same measurement instrument as used in the present study (Frederick et al., 2008), United States women perceived themselves as the smallest compared to Chinese and Croatian women; whereas their perception of the ideal figure was bigger compared to Chinese preference and smaller compared to Croatian preference. Also, they were more satisfied with their body than Chinese women but less satisfied than Croatian women, which is in line with existing studies showing that people from affluent and westernized Asian countries are more dissatisfied with their bodies than people from the United States; while Americans are less happy with their bodies than Europeans or Australians (Holmqvist and Frisén, 2010). This could be explained by the fact that most of the imposed ideals are Eurocentric, which means that women from these countries are more likely to closely fit the ideals compared to Asian populations or countries with greater ethnic diversity, such as the United States. The observed results could also be partially explained from the evolutionary perspective suggesting that men’s view of the attractive body is an important determinant of body image satisfaction among heterosexual women (Bergstrom et al., 2004). Interestingly, women from both countries revealed a preference for thinner figures than the ones selected as the most attractive by the opposite sex, which was consistent with previous studies suggesting that media alter the ideal female body, which women in turn erroneously interpret as the most desirable to men (Sanderson et al., 2002). Similarly, men from both countries selected significantly smaller body size as the one that women from their countries would prefer to have. It is possible that men from both countries thought women wanted such thin bodies, given that women are universally known to diet more (Morry and Staska, 2001) and are more encouraged to diet. Also, women’s magazines tend to display much more ads and articles encouraging weight loss compared to men’s magazines, while, over 75% of the magazine covers for female audience comprise at least one message about how to change the physical appearance (Malkin et al., 1999). Furthermore, Chinese men reported to prefer women of smaller size compared to Croatian men, even though men from both countries were in agreement on the female body size most widely represented in the media, which further suggested that both cultural contexts were exposed to similar thin-ideal media messages, but that thin ideal was endorsed to the higher context in China. Not that long ago, cultures with enough resources had a higher preference for slender bodies and lower body mass index, whereas cultures with scarcer resources had a strong preference for heavier body types (Marlowe and Wetsman, 2001; Swami and Tovée, 2005). Recent research has challenged this type of categorization, proposing that distinctions made based on this cultural basis and distinctions are no longer true and that it is more appropriate to focus on sites’ social, economic status discrimination. That is to say that socioeconomically developed sites have fully adopted thin ideal as a direct result of global westernization (Swami, 2015). Westernized images and products have become especially powerful in non-Western contexts precisely because of their perceived “exclusivity” (Mazzarella, 2003), and those who found themselves between the rapidly changing traditional and modernized world might experience internal uncertainty and identity crises, which in turn might have adverse consequences on their perception and experience of their own body (Nasser et al., 2003). This was further supported with our Study 2, which confirmed how exposure to thin images negatively affected body image satisfaction in women from both cultures, which was also consistent with numerous previous studies (Wegner et al., 2000; Brown and Dittmar, 2005; Harper and Tiggemann, 2008). The observed differences were more prominent in Chinese sample which was in line with the results from Study 1 and Study 2, where levels of thin internalization did significantly vary between two examined cultures; however, they were still universally present in both of the examined samples leading to increased levels of body image dissatisfaction.

Our results provide the basis for a more comprehensive understanding of body image dissatisfaction in China and Croatia. While Croatian women appeared to be relatively satisfied with different aspects of their body, their discontent was relatively consistently expressed across both measures used to evaluate body image size. However, in Chinese women, the discontent with body weight appeared to be less prominent when participants were asked about other physical features, and more evident when participants were asked to focus exclusively on body size. This might be related to variations in cognitive processing styles considering that Chinese people tend to have holistic thinking style (Varnum et al., 2010), and it is possible that when asked to focus on different body parts and facial features, the later ones came to prominence as more important for the overall perception of body image. Contrary, it is possible that thin ideal has already become a normative source of discontent among Chinese women, where discontent with other physical features is emerging as a potentially more problematic matter. The results from the present study could be used to design different counseling programs that could be delivered to women of different age groups. The reported results could also be used by counselors to develop different scales and evaluation instruments, keeping in mind the specific cultural differences of each population, such as the overall tendency of Chinese participants to be more self-critical, as well as differences in thinking styles that could be relevant when selecting the scales to evaluate the body image. Furthermore, women should be explicitly educated about the potential impact of consistent media exposure on body image. They should also be provided education on media literacy, which would allow them to critically analyze the content they are exposed to on a daily basis. In fact, critical screening of the media, and especially online media, appears to be of great importance, especially if we know that an average person spends approximately 6 h per day on the Internet^5^. Finally, parents should be educated on the effect their behavior may have on their child’s perception of their own body. They should limit the “fat talk,” avoid dieting discourses in front of their children, and in general, they should teach their children to focus on the functionality and health of their bodies, as opposed to the looks. The protective role of the family might be especially important for Chinese women since when activating the need for belonging, which previous studies have shown to be related with self-esteem, and approval or support from others (Strudwicke, 2000), Chinese women are more likely to focus and count on relational collectivism. Individuals who internally identify with a culture see their cultural group membership as an integral part of who they are. Accordingly, they are more likely to engage in events and behaviors that are related to their culture (Yeh and Huang, 1996). These results encourage future researchers to expand upon this current study to give voice to unrepresented samples in the body image literature and to develop research of clinical utility for practitioners committed to providing culturally-sensitive services to those susceptible or affected by negative body image.

### Limitations

This study has some limitations that need to be pointed out. First, the present work did not include the Western sample. While the comparison of European and Asian populations provided valuable information, the inclusion of Western samples would provide useful information as well as a valuable reference point for levels of body image dissatisfaction, given that thin-ideal initially originated in industrialized countries. Another limitation refers to the self-report format of the questionnaires, where observed findings may be influenced by a variety of factors such as participant’s feelings or social desirability. For example, body image is often a sensitive topic, and participants may feel too ashamed to report their actual opinions or private details. Thus, there is no way of knowing if reported accounts truthfully represent participants’ attitudes and feelings relating to appearance and body image. Existing studies have shown that Eastern cultures tend to be more negative in self-evaluations (Sedikides et al., 2003), and more self-critical (Heine et al., 2000), which might have a certain effect in this line of research, and thus should be considered by future studies. Finally, Study 1 was a cross-sectional study, and causal relationships could not be inferred, which was partially addressed by Study 2; nonetheless, future experimental studies are necessary to further verify our findings.

## Conclusion

The present research study explored variables related to body image dissatisfaction among Croatian and Chinese women. Our findings indicated that thin internalization could predict body image dissatisfaction in two transition economies, China and Croatia. Besides, Chinese women were, in general, more dissatisfied with their bodies.

## Data Availability Statement

The data that support the findings of this study are available from the corresponding author, XR, upon reasonable request.

## Ethics Statement

This study was carried out in accordance with the recommendations of the Institutional Review Board of the Institute of Psychology, Chinese Academy of Sciences with written informed consent from all subjects. All subjects gave written informed consent in accordance with the Declaration of Helsinki. The protocol was approved by the Institutional Review Board of the Institute of Psychology, Chinese Academy of Sciences.

## Author Contributions

IS conceived the presented idea and developed the theory and wrote the manuscript. XD and IS collected and analyzed the data for Studies 1 and 2. XR and IS collected and analyzed the data for Study 3. All authors conceived and planned the experiments, while XR and IS carried out the experiments. All authors discussed the results and contributed to the final manuscript. XR supervised the project.

## Conflict of Interest

The authors declare that the research was conducted in the absence of any commercial or financial relationships that could be construed as a potential conflict of interest.

## Footnotes

1 https://www.hofstede-insights.com/product/compare-countries/

2 http://hdr.undp.org/en/content/gender-inequality-index-gii

3 www.surveymonkey.com

4 www.wjx.cn

5 www.statista.com
