# Supplementary material for: Body Image and Sociocultural Predictors of Body Image Dissatisfaction in Croatian and Chinese Women
Source: Front Psychol. 2020 May 6;11:731. doi: 10.3389/fpsyg.2020.00731 (PMC7218091; doi:10.3389/fpsyg.2020.00731)

Supplementary material

Contour Rating Scale

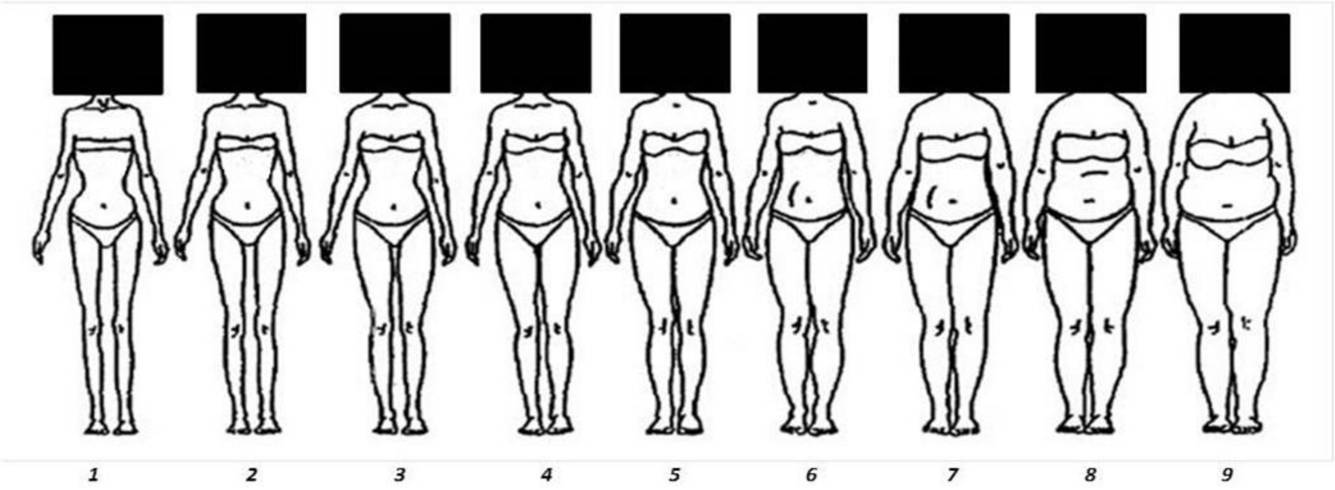

Modified version of Contour Rating Scale

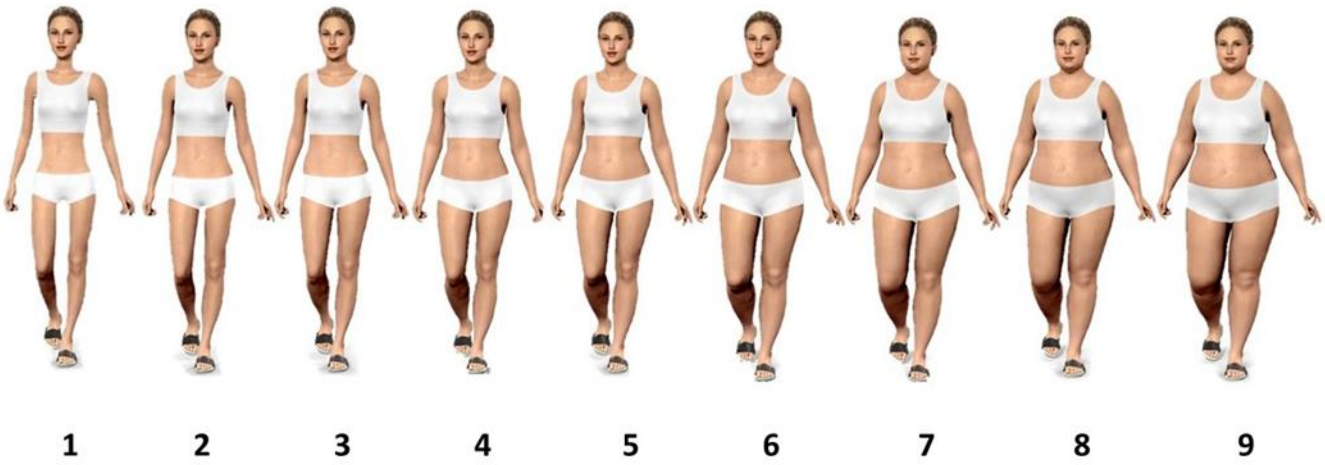

Composite of photos used in the experimental group

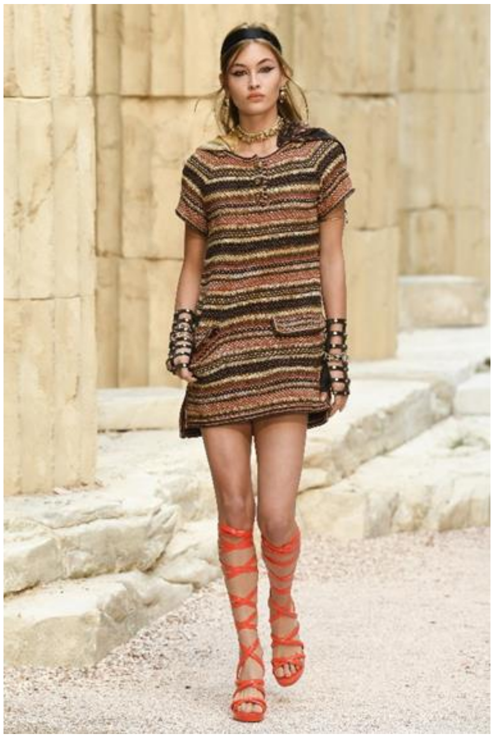

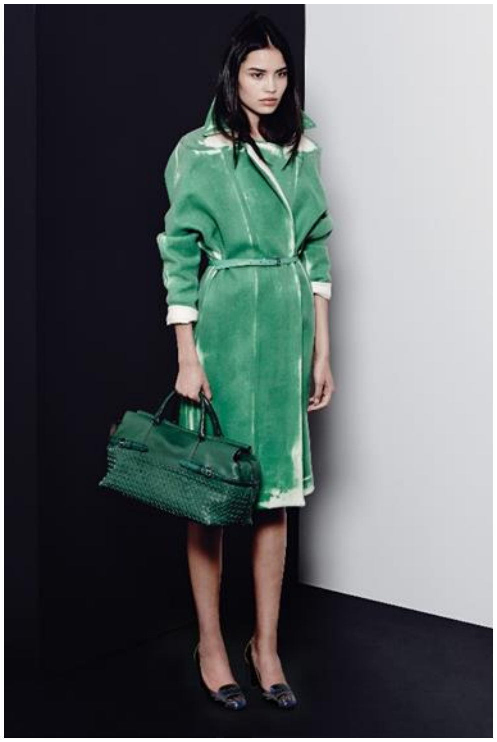

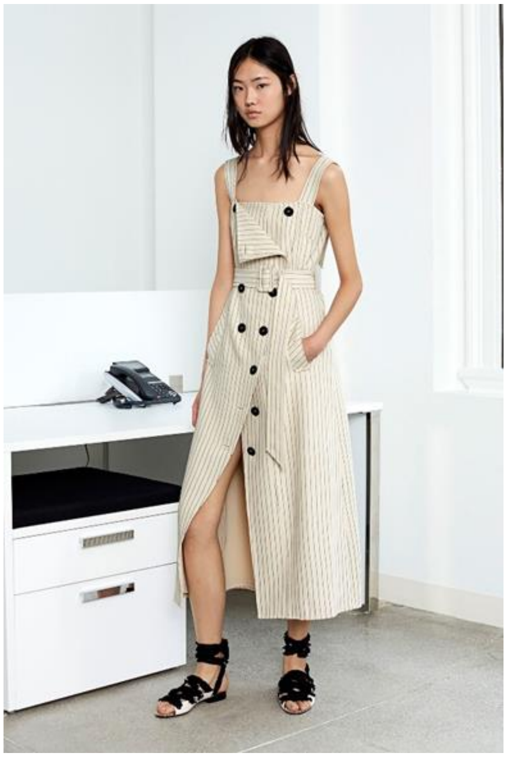

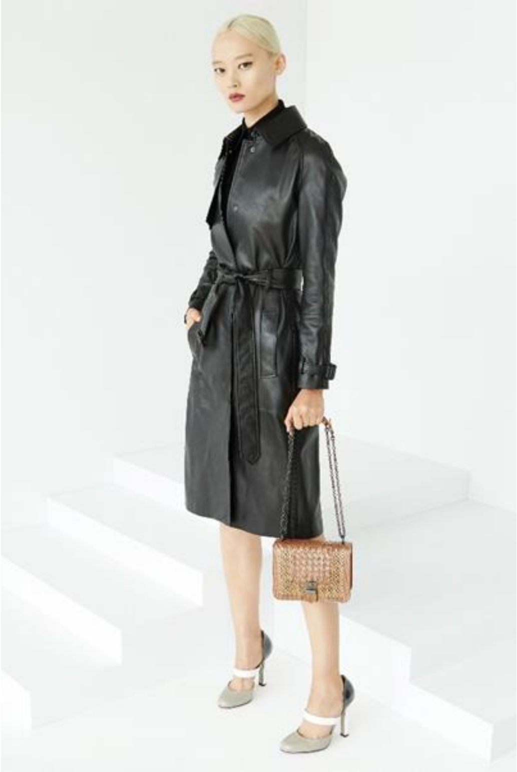

Composite of photos used in the control group

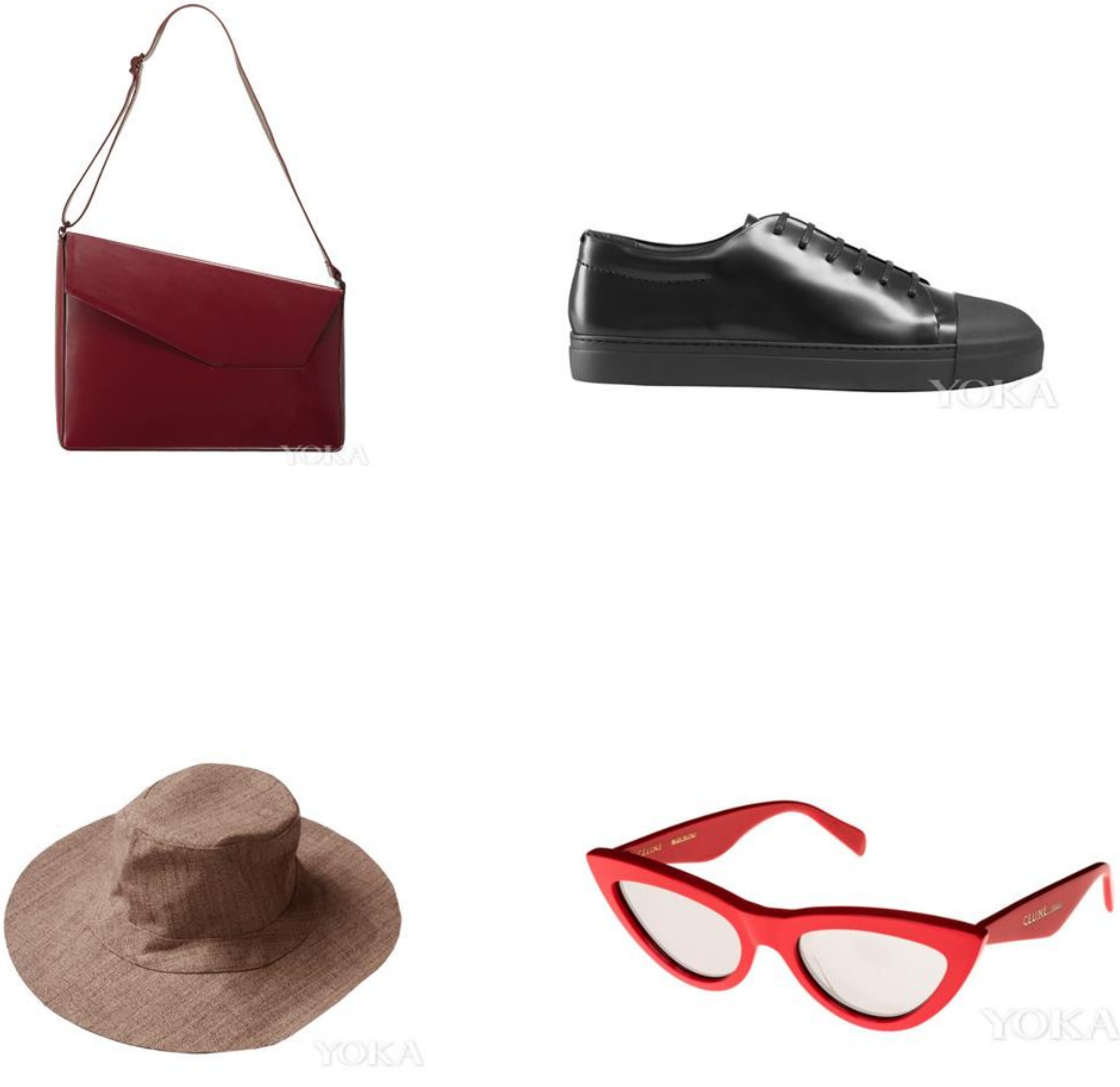

Supplement: Supplementary file 1 [file Image_1.pdf]
